# Cognitive Training with Older Adults Using Smartphone and Web-Based Applications: A Scoping Review

**DOI:** 10.14283/jpad.2024.17

**Published:** 2024-01-23

**Authors:** A. F. Silva, Rui Miguel Silva, E. Murawska-Ciałowicz, G. Zurek, N. Danek, M. Cialowicz, J. Carvalho, F. M. Clemente

**Affiliations:** 1 Escola Superior Desporto e Lazer, Instituto Politécnico de Viana do Castelo, Rua Escola Industrial e Comercial de Nun’Álvares, 4900-347 Viana do Castelo, Portugal; 2 Research Center in Sports Performance, Recreation, Innovation and Technology (SPRINT), 4960-320 Melgaço, Portugal; 3 The Research Centre in Sports Sciences, Health Sciences and Human Development (CIDESD), 5000-801 Vila Real, Portugal; 4 Department of Physiology and Biochemistry, Wroclaw University of Health and Sport Sciences, 51-612 Wrocław, Poland; 5 Department of Biostructure, Wroclaw University of Health and Sport Sciences, 51-612 Wrocław, Poland; 6 Department of Physiotherapy in Interventional Medicine and Oncology, Wroclaw University of Health and Sport Science, 51-612 Wrocław, Poland; 7 High Performance Brain, Corkstraat 46, 3047 AC Rotterdam, Netherlands; 8 Gdansk University of Physical Education and Sport, 80-336 Gdańsk, Poland; 9 Escola Superior de Desporto e Lazer, Instituto Politécnico de Viana do Castelo, Complexo Desportivo e Lazer Comendador Rui Solheiro Monte de Prado, 4960-320 Melgaço, Portugal

**Keywords:** Cognition, mild cognitive impairment, Alzheimer’s disease, mobile tech, cognitive training

## Abstract

**Introduction:**

The present scoping review focused on: i) which apps were previously studied; ii) what is the most common frequency for implementing cognitive training; and iii) what cognitive functions the interventions most focus on.

**Methods:**

PRISMA guidelines were followed, and the search was conducted on Web of Science, PsycInfo, Cochrane, and Pubmed. From 1733 studies found, 34 were included.

**Results:**

it was highlighted the necessity for forthcoming investigations to tackle the methodical restrictions and disparities in the domain.

**Discussion:**

great diversity in intervention protocols was found. Incorporating evaluations of physical fitness in conjunction with cognitive evaluations can offer a more all-encompassing comprehension of the impacts of combined interventions. Furthermore, exploring the efficacy of cognitive training applications requires additional scrutiny, considering individual variances and practical outcomes in real-life settings.

## Introduction

**D**ementia, a progressive neurocognitive disorder, is characterized by the deterioration of cognitive functions encompassing language, memory, perception, and thought, persisting until an individual’s passing (1). A diagnosis of dementia is made when two or more of these core mental functions are impaired (2). As global populations continue to age, the prevalence of dementia is increasing, with current estimates indicating that approximately 44 million people are affected by this chronic neurological condition (3). Projections indicate that by 2050, this chronic neurological condition will afflict approximately 14,298,671 individuals in the European Union (EU) and 18,846,286 in the broader European region (4). Notably, among the elderly population, 50% to 80% of individuals aged 70 or older, scoring within the normal range on cognitive tests, report perceived cognitive decline (2).

According to the National Institute of Aging-Alzheimer’s Association (NIA-AA) (5), Alzheimer’s disease, the most common form of dementia, exhibits a symptomatology that spans six progressive stages, reflecting the increasing severity of the condition. In light of this, there appears to be a window of opportunity for delaying the development of dementia, especially considering the absence of a known cure. Recognizing this, the World Health Organization (WHO) has designated dementia as a public health priority. Between a healthy state and the diagnosis of dementia lies a condition known as mild cognitive impairment (MCI). Individuals with MCI also experience cognitive declines, but they typically maintain their independence and continue to function well in everyday life (6, 7). These cognitive declines typically affect processing speed, executive functions, memory, and visuospatial abilities (8).

In response, the concept of cognitive training and stimulation has evolved. Cognitive training involves repetitive practice targeting specific cognitive functions with standardized tasks of varying difficulty, while cognitive stimulation takes a non-specific approach, emphasizing diverse activities and social interaction (9, 10). Fortunately, interventional studies on both cognitive training and cognitive rehabilitation have shown promising effects in improving cognitive function among individuals with MCI (11–14). This is attributed to the human brain’s capacity for neuroplasticity, which refers to the brain’s ability to undergo morphological changes in response to environmental stimuli (15). Consequently, the brain can adapt and compensate for cognitive changes by reinforcing existing connections or forming new ones. This capacity persists throughout one’s lifespan and can be influenced by various factors, including genetics, education level, occupation, socioeconomic status, physical health, lifestyle choices, and mental engagement (16).

Technology’s ubiquity, even among older adults, has resulted in numerous smartphone and/or web-based applications (Apps) designed to assist individuals with MCI. Within the realm of cognitive training and stimulation, studies have identified promising strategies for preserving cognitive function in healthy older adults and individuals with MCI (17). Comparing technology-based interventions with traditional programs has shown the former to yield overall superior outcomes in enhancing cognitive function and quality of life (18–20). Computerized cognitive training’s beneficial effects endure in individuals with preserved cognitive function, both short-term and long-term (21). Additionally, cognitive training has been recognized as one of the most effective interventions, as it has the potential to decelerate the progression of cognitive decline in individuals with dementia in rehabilitation settings (22, 23). However, while technology-based cognitive training interventions hold promise, a systematic review revealed that their effectiveness remains inconsistent, primarily due to variations in study design (24).

As there is currently no known cure for dementia, prioritizing the enhancement of preventive strategies to delay its onset and slow down cognitive decline has become imperative (12). Furthermore, the pharmaceutical treatments currently used for dementia do not consistently produce significant modifications in the progression of the disease (25). As a result, the cognitive training approach has garnered considerable attention (26, 27). For those reasons, the present scoping review had three main objectives, which were to describe the apps previously studied, investigate the most common frequency for implementing cognitive training, and assess the primary cognitive functions targeted by these interventions.

## Methods

### Protocol and registration

This scoping review followed the PRISMA 2020 guidelines (28) and also took into consideration the recommendations for the scoping reviews checklist (PRISMA-ScR) (29). The protocol was pre-registered in the Open Science Framework (OSF) (associated with the project: https://osf.io/e4znf/).

### Eligibility criteria

Studies published in peer-reviewed journals, including those with the status of “in press” or “ahead-of-print”, were considered. No limitations to date were established. Only English studies were included. The eligibility criteria were determined following the PECOS approach (population, exposure, comparator, outcome, study design): (i) population: inclusion of cognitively healthy older adults and older adults with cognitive impairment (including Alzheimer’s, dementia, or other cognitive declines); (ii) exposure: participants exposed to cognitive training, including technology-based methods (web-based apps and smartphone apps), as well as manual games/tasks; (iii) comparator: individuals not exposed to medication; (iv) outcome(s): assessment of the implemented games/tasks and the specific cognitive functions targeted; and v) study design: consideration of both observational studies and interventions.

### Information sources

The present study used the following databases Web of Science, PsycInfo, Cochrane, and Pubmed. After performing the protocol registration, the searches were conducted on the same day. In addition to the database searches, a manual search was performed on: (i) the reference lists of included studies to identify potentially relevant titles and checking the abstract for pertinent inclusion criteria and, if necessary, the full text; (ii) snowballing citation tracking, preferably in Web of Science; and (iii) consultation of two external experts (as recognized by Expertscape: https://expertscape.com/ex/soccer). Finally, errata/retractions will also be analyzed for any articles that were included (30).

### Search strategy

The search strategy applied the Boolean operators AND/OR. No filters or limitations were used (e.g., date; study design) to maximize the chances of identifying appropriate studies (31). The main search strategy was as follows:

[All fields/Full text] “cogni* rehabilitation” OR “cogni* stimulation” OR “cogni* train*”

AND

[All fields/Full text] Alzheimer OR “cogni* decline” OR “cogni* impair*” OR MCI OR dementia

AND

[All fields/Full text] software* OR “mobile app*” OR “smartphone app*” OR “smartphone applications*” OR “mobile tech*” OR “assistive tech*”

Table 1 presents the codes used for each database.

**Table 1:** Resulting code lines for each database (preliminary tests, before registering the protocol; no records were retrieved at this point)

**Database**	**Specificities of each database**	**Code lines after performing the search**
Web of Science	Title, abstract and keywords (termed “Topic”).	(“cogni* rehabilitation” OR “cogni* stimulation” OR “cogni* train*”) AND (Alzheimer OR “cogni* decline” OR “cogni* impair*” OR MCI OR dementia) AND (software* OR “mobile app*” OR “mobile tech*” OR “assistive tech*” OR “virtual realit*” OR “augmented reality”)
PsycInfo	Nothing to report	(Any Field: cogni* rehabilitation OR Any Field: cogni* stimulation OR Any Field: cogni* train*) AND (Any Field: Alzheimer OR Any Field: cogni* decline OR Any Field: cogni* impair* OR Any Field: MCI OR Any Field: dementia) AND (Any Field: software* OR “mobile app*” OR “mobile tech*” OR “assistive tech*” OR “virtual realit*” OR “augmented reality”)
Cochrane	Title, abstract and keywords	“cognit* rehabilitation” OR “cognit* stimulation” OR “cognit* train*” in Title Abstract Keyword AND Alzheimer OR “cogni* decline OR cogni* impair* OR MCI OR dementia in Title Abstract Keyword AND software* OR “mobile app*” OR “mobile tech*” OR “assistive tech*” OR “virtual realit*” OR “augmented reality” in Title Abstract Keyword — (Word variations have been searched)
Pubmed	Nothing to report	(“cogni* rehabilitation” OR “cogni* stimulation” OR “cogni* train*”) AND (Alzheimer OR “cogni* decline” OR “cogni* impair*” OR MCI OR dementia) AND (software* OR “mobile app*” OR “mobile tech*” OR “assistive tech*” OR “virtual realit*” OR “augmented reality”)

### Selection process

Two of the authors (AFS and JC) independently retrieved records, which included titles and abstracts. These same authors then conducted an independent screening of the collected full-text articles. Any disagreements between the two authors were resolved through joint reanalysis. In cases where a consensus could not be reached, a third author (FMC) made the final decision. Whenever necessary, all co-authors provided their input on any uncertainties that arose during the selection process to support the final decision. Mendeley software (Elsevier) was utilized for managing records, including the automated or manual removal of duplicates.

### Data collection

The primary author (AFS) initially conducted the data collection process. Subsequently, two co-authors (ND and MC) performed a double-check to ensure the accuracy and completeness of the collected data. To facilitate this process, a specially designed Microsoft® Excel datasheet was created and used for data extraction, capturing essential information. The Excel datasheet is available in the supplementary material for reference. In cases where relevant data were missing from the full text, the primary author (AFS) directly contacted the corresponding author of the document via email and/or ResearchGate to obtain the necessary information.

### Data items

In this section, the data items considered for the review are detailed. These included participant characteristics such as age, sex, cognitive training frequency, and the stage of dementia. Context-related information, specifically the number of sessions under consideration, was also captured. The main outcomes of interest encompassed the tests that were implemented and the cognitive functions assessed. Comparisons were made with other cognitive interventions, which might have involved medical or musical interventions, or a control group undergoing any form of intervention. Additionally, supplementary information was recorded, including the publication date, funding details, and any competing interests disclosed by the studies under review.

### Study risk of bias assessment

Two authors (EMC and JC) independently assessed the risk of bias. In the case of disagreements, both reanalyzed the process. In the case of no subsequent consensus being reached, a third author (AFS) made the final decision. The Risk of Bias Assessment Tool for Nonrandomized Studies (RoBANS) was used to assess the risk of bias in the included studies (32). The scale has shown moderate reliability, feasibility, and validity (32). The tool comprises six domains: (i) the selection of participants; (ii) confounding variables; (iii) the measurement of exposure; (iv) the blinding of the outcome assessments; (v) incomplete outcome data; and (vi) selective outcome reporting. The domains are classified as ‘low’, ‘high’, and ‘unclear’ risk of bias.

### Data management and synthesis methods

A narrative synthesis was performed, including data summaries presenting numbers and percentages for predefined data items. To provide an overview of the existing research landscape and emphasize areas requiring further investigation, an evidence-gap map (EGM) was developed. The EGM serves as a visual representation to convey the existing evidence and highlight current research gaps (33–35). Table 2 presents a provisional EGM.

**Table 2:** Evidence-gap map

**Author (year)**	**Software**	**Sample size**	**Sex**	**Cognitive training frequency**	**Stage of dementia**	**Duration of the exposure**	**Cognitive function covered**
Wolinsky et al., 2013	Road Tour	681	Unreported	5 per week for 2 h	Healthy old adults	10h	Attention
Wolinsky et al., 2011	Road Tour	681	Unreported	10h	Healthy old adults	10h	Attention
Rai et al., 2021	iCST app	61	Male: 42 Female: 19	2-3 per week for 30 min or more	Mild to moderate dementia	11 weeks	Executive functions, attention, memory
Rai et al., 2021	iCST app	30	Unreported	2-3 per week for 30 min	Mild to moderate dementia	11 weeks	Executive functions, attention, memory
Gambella et al., 2022	Brainer	78	Unreported	2 per week for 60 min	Alzheimer	8 weeks	Attention, executive functions, memory, language, and praxis abilities
Cavallo et al., 2016	Brainer	80	Unreported	3 per week for 30 min	Alzheimer	12 weeks	Visuospatial, attention, language, executive functions, memory, praxis abilities.
Cavallo & Angilletta, 2019	Brainer	40	Unreported	3 per week for 30 min	Alzheimer	12 weeks	Attention, language, executive functions, memory, praxis abilities
Nousia et al., 2019	RehaCom	25	Males: 6 Females 19	2 per week for 60 min	MCI	15 weeks	Memory, attention, and executive function
Bahar-Fuchs et al., 2019	CogniFit	85	Male: 51 Female: 34	3 per week, 2 sessions on each training day, for 10–15 min	At risk: diagnosis of type 2 diabetes but no diagnosis of dementia or Alzheimer’s disease.	8 weeks, 48 sessions	Memory, attention, praxis abilities and executive functions
Bahar-Fuchs et al., 2017	CogniFit	45	Male: 15 Female: 10	3 per week for 20–30 min	MCI	8–12 weeks	Memory, attention, praxis abilities, and executive functions
Gigler et al., 2013	CogniFit	7	Male: 3 Female: 4	2 per week for 30 min	MCI	8–10 weeks, 17 sessions	Memory, attention, praxis abilities, and executive functions
Beishon et al., 2019	Lumosity	60	Unreported	5 per week for 30 min	MCI and AD	12 weeks	Attention, memory, visuospatial, and language.
Robert et al., 2020	MeMo	25	Male: 15 Female: 10	4 per week	Diagnosed with neurocognitive disorders	12 weeks	Memory, attention, and executive functions
Fernández-Calvo et al., 2011	Big Brain Academy	30	Male: 17 Female: 13	3 per week for 60 min	MCI	12 weeks	Attention, memory, executive functions
Haesner et al., 2015	LeVer	40	Male: 8 Female: 32	120 min	Subjective memory impairment	8 weeks	Memory, language, executive functions
Assis et al., 2009	Geriatric	1	Male	2 per week for 50 min	Alzheimer	4 months	Orientation, language, attention, memory, and executive functions
Ng et al., 2021	ProAge	96	Male: 13 Female: 83	2 per week	At risk for cognitive impairment	24 weeks	Memory, and executive functions
Meltzer et al., 2023	BrainHQ and Duolingo	BrainHQ: 24 Duolingo: 28	Male: 7 Female: 17 Male: 10 Female: 18	5 per week for 30 min	Healthy old adults	16 weeks	BrainHQ: Attention, memory, and executive functions Duolingo: language
Eckroth-Bucher & Siberski, 2009	Captain’s Log	15	Male: 1 Female: 14	2 per week for 45 min	Mild to moderate dementia	6 weeks	Executive functions
Corbett et al., 2015	ReaCT and GCT	ReaCT: 2557 GCT: 2432	Male: 1561 Female: 3428	daily, 10 min	Old adults (do not mention if they have dementia)	6 months	ReaCT: executive functions GCT: executive functions, attention, memory, and visuospatial
Nicholson et al., 2022	Useful Field of View Training (UFOVT)	Unreported	Unreported	2-3 sessions per week until 525 levels	MCI or dementia	6 months	Attention
Suo et al., 2016	COGPACK	100	Male: 32 Female: 68	2 per week for 90 min	MCI	26 weeks	Attention, memory, language
Singh et al., 2014	COGPACK	51	Unreported	2-3 per week for 75 min	MCI	6 months	Memory, attention, executive functions
Hwang et al., 2015	COMCOG	35	Male: 14 Female: 21	5 per week for 30 min	Alzheimer	4 weeks	Memory and language
Hagovská et al., 2017	CogniPlus	30	Male: 12 Female: 18	2 per week for 30 min	MCI	10 weeks	Attention, executive functions, memory, and praxis abilities
Gaitán et al., 2013	FesKits	37	Male: 15 Female: 8	2-3 times per week	MCI and AD	12 weeks, 30 sessions	Attention, executive functions, and memory
Barban et al., 2016	SOCIABLE	301	Male: 129 Female: 172	2 per week for 30 min	MCI and AD	3 months	Memory, executive functions, orientation, language, and praxis abilities
Laiz et al., 2018	NeuronUp	32	Male: 14 Female: 18	20 min	MCI	5 weeks, 5 sessions	Memory
Vermeij et al., 2017	Cogmed	14	Male: 10 Female: 4	5 per week for 45 min	MCI	5 weeks, 25 sessions	Executive functions
Vermeij et al., 2015	Cogmed	18	Male: 14 Female: 4	5 per week for 45 min	MCI	5 weeks, 25 sessions	Executive functions
Hyer et al., 2016	Cogmed	34	Male: 17 Female: 17	25 sessions for 40 min	MCI	5-7 weeks	Executive functions
González-Palau et al., 2014	GRADIOR	11	Unreported	3 per week for 40 min	MCI	12 weeks	Attention and memory
Vanova et al., 2018	GRADIOR and ehco-BUTLER	GRADIOR: 85 ehcoBUTLER: 85 Both: 85	Unreported	3-4 per week for 30 min	Dementia and MCI	12 months	GRADIOR: Attention, memory, language, visuospatial, executive functions, and praxis abilities ehcoBUTLER: Attention, memory, language, visuospatial, executive functions
De Luca et al., 2016	Unreported	20	Males: 10 Females: 10	3 per week	MCI	8 weeks, 24 sessions	Attention, memory, language, and praxis abilities

MCI: mild cognitive impairment; AD: Alzheimer’s disease

## Results

### Literature search

**Figure 1:**
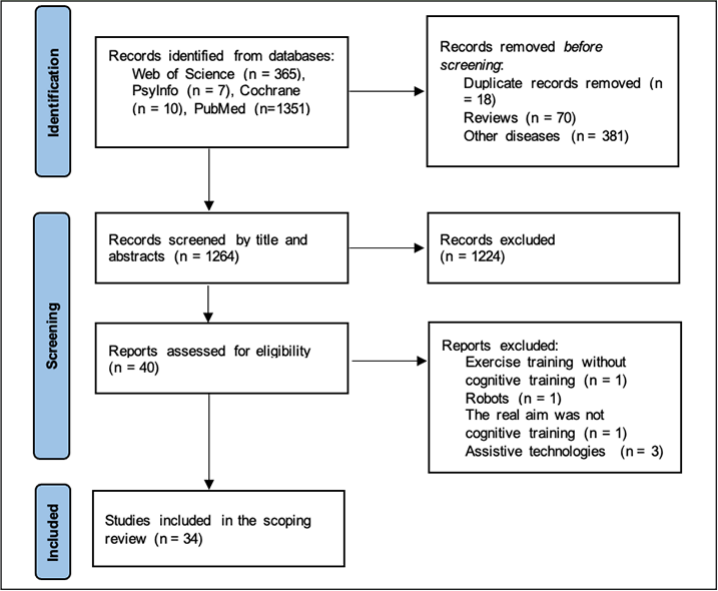
PRISMA flow diagram

The literature search showed the existence of 1733 studies. From those, 469 were excluded as they were duplicated (n = 18), others were reviews (n = 70), and the majority of them were from other diseases such as Parkinson’s, Schizophrenia, cancer, and strokes, among others (n = 381). In screening the title and abstract, 1224 were excluded as they focused on other rehabilitation strategies/protocols or did not have any technology implemented. Moreover, other articles did not include interventions and just presented an app with only one applied session. In the end, 40 studies were assessed for eligibility by full-text analysis. Finally, 34 articles met the criteria for inclusion. A flowchart of the literature search and selection process is presented in Figure 1.

### Apps used

In the 34 studies included, 27 apps were used. The Brainer, CogniFit, and Cogmed were the most used apps with 3 studies published for each app, followed by Road Tour, iCST app, COGPACK, and GRADIOR with 2 studies published for each app. The other studies had only one study published.

### Study characteristics

The studies varied greatly in the characteristics of the sample, with studies from just one subject (Assis et al., 2010) including up to 2557 subjects (36). Nevertheless, from the 34 studies included, only 4 studies integrated 100 or more subjects in their samples. The other studies presented a mean of approximately 39 subjects.

Most of the studies (65%, corresponding to 22 studies) included both sexes in the interventions, and only one study included a very small sample size, consisting of one male. However, the remaining 32% of the studies (11 studies) did not describe the sex of the subjects included in the sample. They only mentioned that they were old adults or adults. Considering the stage of dementia, 3 studies did not include any dementia or impairment (37–39), and one study did not mention any information regarding the state of mental and cognitive health (36), and 2 other studies analyzed subjects at risk of dementia (40, 41). The remaining 28 studies included subjects with mild to moderate impairment or even with a diagnosis of Alzheimer’s disease.

### Types of interventions

The duration of the interventions was quite varied, with a range of 20 (42) to 600 (37, 38) minutes per week of practice in the technologies, with a range of 10 (36, 40) to 240 minutes per session. Also, the frequency varied between 2 (18, 41, 43–53) to every day (36) (of all the studies that explicitly mentioned the frequency) and lasted from just one week with 10 intensive hours (37, 38) to 12 months (54).

### Cognitive functions covered

Considering all studies, the cognitive functions covered were memory (79.4%), attention (73.5%), executive functions (76.5%) language (35.3%), praxis abilities (26.5%), visuospatial (11.8%), and orientation (5.9%). The cognitive functions of memory, executive functions, and attention stand out clearly with 27, 26, and 25 studies, respectively.

## Discussion

This scoping review aimed to provide insights into the landscape of cognitive training apps, their implementation frequencies, durations, and the primary cognitive functions they target. While our analysis shed light on these aspects, it is equally important to delve into the distinctive challenges and nuances associated with the use of these apps in the context of older adults living with cognitive impairment. This expanded discussion aims to address these specific considerations.

Most of the protocols and interventions used to assess cognitive functions focused mainly on memory, executive functions, and attention tasks. Among others, the most used apps during cognitive function training interventions were the Brainer, CogniFit, and Cogmed apps. Although the majority of these studies assessed cognitive impairments through the use of questionnaires, such as the mild cognitive impairment Peterson criteria (55), the Mini-mental state (56), the Alzheimer’s disease assessment scale (57), and the Cornell scale for depression in dementia (58), the overall studies did not categorize their samples according to each cognitive impairment that was assessed at the baseline. Different cognitive impairments show differentiated characteristics and may respond differently to cognitive training interventions (12). Without accounting for these differences, it may be challenging to draw accurate conclusions about the effectiveness of apps in addressing specific cognitive impairments.

The wide variation in intervention protocols, such as the type, duration, intensity, and frequency of cognitive training, may lead to inconsistent findings and limit the ability to compare and generalize the results. For instance, while the overall studies implemented an average of two sessions per week (18, 45, 47, 51, 59, 60), one study implemented cognitive training sessions on all days of week (61). Also, the duration of cognitive training was widely widespread, from a minimum of 20 minutes to a maximum of 600 minutes per week (37, 38, 42). Additionally, it is worth noting that 10 of the 34 studies analyzed (37, 51, 60, 62–68) did not specify the sex composition of their samples. Neglecting to provide information regarding the sex distribution within these studies may introduce biases that hinder the comprehensive understanding of their findings and impede meaningful comparisons with the existing evidence.

Moreover, there are other challenges associated with the use of these apps that must be addressed. For instance, as individuals age, there is a natural decline in cognitive function, including a decrease in processing speed (69). This highlights the importance of considering the baseline cognitive abilities and age-related changes when implementing cognitive training apps for older adults with cognitive impairment. The literature suggests that basic numerical skills are generally preserved in healthy aging (70). However, individuals with MCI show difficulties in understanding numerical and arithmetic tasks (71). This finding indicates that when designing cognitive training apps, it’s essential to distinguish between age-related changes and impairments specific to cognitive conditions such as MCI. Importantly, the impact of cognitive training apps may vary based on individual experience and perceptions (72). Given that, it is imperative to match the technology to older adults’ needs and assess their experiences. Lastly, although early detection of cognitive decline can aid research and clinical intervention, it poses adherence challenges to the use of such smartphone/web-based cognitive training apps (73).

The present scoping review has certain limitations that warrant attention. One limitation pertains to the employed search strategy and criteria for article eligibility. We implemented a search strategy that was unanimously agreed upon by our team of authors and experts. Nonetheless, it is important to acknowledge that like any search strategy, it may not have captured all relevant articles. Nonetheless, we made efforts to minimize this potential bias by employing a comprehensive search strategy. Our research methodology was conducted by the PRISMA statement, ensuring a comprehensive and rigorous approach that surpasses traditional scoping reviews. A previous systematic review which adroitly harnessed between-study differences to investigate factors such as dosage effects and target-specific outcomes, offers a noteworthy path forward in this domain (24). The mentioned systematic analysis highlighted the existence of modest yet discernible efficacy of computerized cognitive training in enhancing cognitive performance among healthy older adults, all while delineating the influential role played by design factors (24). Although this approach was out of the scope of this scoping review, our presentation of results was guided by a rationale that prioritized the analysis of methodological approaches utilized in the original studies, expanding beyond the sole consideration of primary outcomes reported.

Further research is essential to determine which cognitive training interventions work best for different populations and cognitive domains. Standardizing protocols and comparing various approaches, such as computer-based training, gamified interventions, and combined interventions with physical training, can help address these questions. Future research should identify factors influencing responses to cognitive training, including age, baseline cognitive abilities, genetic predispositions, and individual differences. Moreover, longitudinal studies could provide insights into the long-term effects of combined cognitive and physical interventions on cognition, fitness, and well-being. Future research may explore personalized cognitive interventions through apps and physical training, tailoring exercises based on participants’ cognitive abilities and fitness levels.

## Conclusion

This scoping review highlights the multifaceted challenges in using cognitive training apps for older adults with cognitive impairment. Categorizing participants based on cognitive impairment type, addressing intervention heterogeneity, and considering age-related changes are crucial for effective app-based interventions. Moreover, recognizing individual experiences and needs, assessing adherence issues, and adapting technology accordingly is essential. Future research should focus on standardizing protocols, understanding population-specific responses, and exploring personalized cognitive interventions through apps and physical training for improved cognitive health in older adults.
